# Clinical interest of *KRAS* mutation detection in blood for anti-EGFR therapies in metastatic colorectal cancer

**DOI:** 10.1038/sj.bjc.6604451

**Published:** 2008-07-01

**Authors:** F Di Fiore, F Charbonnier, B Lefebure, M Laurent, F Le Pessot, P Michel, T Frebourg

**Affiliations:** 1 Digestive Oncology Unit, Department of Hepato-Gastroenterology, University Hospital, Rouen, France; 2 Inserm U614, Faculty of Medicine, Institute for Biomedical Research, Northwest Canceropole, Rouen, France; 3 Department of Surgery, University Hospital, Rouen, France; 4 Department of Pathology, University Hospital, Rouen, France

**Sir**,

We reported last year, in the *British Journal of Cancer*, in a series of 59 metastatic colorectal cancer (MCRC) patients treated with cetuximab-based chemotherapy (CT), that *KRAS* mutation was highly predictive of treatment resistance and that progression-free survival was significantly increased in wild-type *KRAS* compared with mutant *KRAS* patients (Di Fiore *et al*, 2007). All the studies published so far have unambiguously confirmed that the presence of somatic *KRAS* mutation is indeed highly predictive of resistance to anti-EGFR antibodies in MCRC patients (Lièvre *et al*, 2006, 2008; Benvenuti *et al*, 2007; Frattini *et al*, 2007; Khambata-Ford *et al*, 2007; De Roock *et al*, 2008). Moreover, a large randomised controlled trial on panitumumab integrating *KRAS* genotyping has recently shown that, among 208 patients receiving panitumumab, 0 out of 84 mutants and 21 out of 124 (17%) wild-type patients were, respectively, responders (Amado *et al*, 2008). Therefore, *KRAS* genotyping should now be performed on a routine basis in patients with MCRC. In most of these studies, *KRAS* genotyping has been performed on primary colorectal tumours, whereas anti-EGFR antibodies are used to treat the metastatic disease. This strategy might, at least in certain circumstances, present two limitations. First, systematic *KRAS* genotyping in MCRC patients might be hampered in the future, at least for some patients, by the difficulty of obtaining tumour samples suitable for molecular analyses (and this might limit the use of anti-EGFR antibodies). Second, considering the genetic heterogeneity of colorectal cancers, the absence of detectable *KRAS* mutations in the primary tumour cannot formally exclude the presence of a *KRAS* mutation in metastases. For these two reasons, we think that detection of *KRAS* mutation in the blood of patients with MCRC may have a clinical interest in the context of anti-EGFR therapies and we would like to highlight in this letter the potential interest of such a strategy. Although several studies have shown the presence of mutant DNA in blood from patients with colorectal neoplasia, only positive results are informative. Therefore, one should consider the development of combined tests indicating in blood, first the presence of tumour DNA, then the status of *KRAS*. In MCRC, hypermethylated DNA can be used as a blood tumour molecular marker. For instance, hypermethylation of the *RASSF2* gene has frequently been detected in colorectal adenoma and invasive carcinoma (Park *et al*, 2007), and we found, in a series of 32 patients with MCRC, that *RASSF2* was hypermethylated in 79% of the tumours (unpublished results). In addition to *RASSF2*, other targets may be used to ensure a sensitive detection of tumour DNA, if *RASSF2* is not found hypermethylated. For sensitive detection on a routine basis of *KRAS* mutation, several methods, shown to be more sensitive than conventional dye-labelled dideoxynucleotide sequencing, are now available, such as, SNaPshot or PCR-LCR assays (Di Fiore *et al*, 2007), or allele-specific real-time PCR (De Roock *et al*, 2008; Lièvre *et al*, 2008). We used this strategy of combined blood assays to analyse two patients who received cetuximab-based CT, one responder and the other showing a progressive disease after anti-EGFR therapy. We screened the plasma of these patients for the presence of methylated DNA, using a classical methyl-specific assay exploring the *RASSF2A* promoter after bisulphite treatment, and then for the presence of *KRAS* mutation using real-time PCR, performed in the presence of a peptide nucleic acid (PNA) sequence specific for the wild-type *KRAS* codons 12 and 13, which inhibits amplification from the wild-type template. The first patient, a 67-year-old man, received cetuximab and irinotecan regimens for a peri-hepatic lymph node tumour recurrence 12 months after surgery for liver metastases, and after 3 months, evaluation revealed disease progression. In patient plasma collected before the beginning of cetuximab CT, the combined assays revealed the presence of hypermethylated *RASSF2* (Figure 1A) and the presence of mutant *KRAS*. Sequencing analysis of the PCR product obtained in the presence of the PNA revealed the same *KRAS* mutation (Figure 1B), as the one previously detected in the colorectal tumour and liver metastases. The second patient, a 76-year-old man, received, in second line, cetuximab plus irinotecan CT for hepatic metastasis occurring 4 years after curative surgery for a bifocal CRC adenocarcinoma, and this treatment allowed control of the disease with the duration of response of 10 months. In this patient, the combined assays performed on the plasma collected before cetuximab treatment showed the presence of hypermethylated *RASFF2A* (Figure 1) but the absence of mutant *KRAS*.

We therefore suggest that, in the forthcoming clinical trials on anti-EGFR antibodies in MCRC, which integrate *KRAS* genotyping, it is probably useful to collect blood samples before treatment and that the clinical interest of such combined blood tests, using the presence of hypermethylated DNA, as tumour DNA marker, and a sensitive method for *KRAS* mutation detection, should be evaluated on large series of MCRC patients.

## Figures and Tables

**Figure 1:**
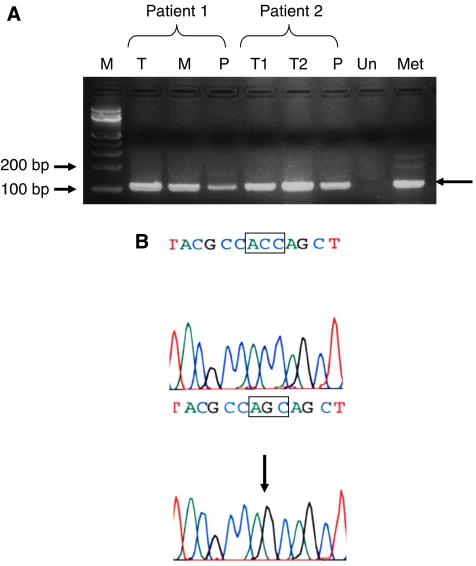
Detection of methylated *RASSF2A* promoter in the primary tumour (T), liver metastases (M) and plasma (P) from patients 1 and 2. For patient 2, T1 and T2 correspond to the right and left colon adenocarcinoma, respectively. Genomic DNA was modified by bisulphite treatment and amplified with primers specific of the methylated *RASSF2A* promoter. M, molecular marker; Un, unmethylated DNA used as a negative control; Met, methylated DNA, used as a positive control. The arrows indicate the 110 bp amplified product (**A**). Detection of a *KRAS* mutation in the plasma from patient 1. Two independent real-time PCRs were performed from DNA extracted from plasma, in the presence and in the absence of a PNA specific of the wild-type *KRAS* sequence. The presence of mutant DNA within the sample is detected by a significant shift towards lower values of the cycle threshold (C_*t*_) when the PNA is added to the reaction. The upper and lower panels correspond to the sequences of the amplified products obtained in the absence and presence of the PNA, respectively. In the presence of the PNA, only the mutant allele is amplified. The sequences correspond to the antisense strand, the box marks codon 12 and the arrows the c.35G>C mutation (**B**).

## References

[bib1] Amado GR, Wolf M, Peeters M, Van Cutsem E, Siena S, Freedman DJ, Juan T, Sikorski R, Suggs S, Radinsky S, Patterson SD, Chang DD (2008) Wild-Type KRAS is required for panitumumab efficacy in patients with metastatic colorectal cancer. J Clin Oncol 26: 1626–1634 18316791 10.1200/JCO.2007.14.7116

[bib2] Benvenuti S, Sartore-Bianchi A, Di Nicolantonio F, Zanon C, Moroni M, Veronese S, Siena S, Bardelli A (2007) Oncogenic activation of the RAS/RAF signaling pathway impairs the response of metastatic colorectal cancers to anti-epidermal growth factor receptor antibody therapies. Cancer Res 67: 2643–2648 17363584 10.1158/0008-5472.CAN-06-4158

[bib3] De Roock W, Piessevaux H, De Schutter J, Janssens M, De Hertogh G, Personeni N, Biesmans B, Van Laethem JL, Peeters M, Humblet Y, Van Cutsem E, Tejpar S (2008) KRAS wild-type state predicts survival and is associated to early radiological response in metastatic colorectal cancer treated with cetuximab. Ann Oncol 19: 508–515 17998284 10.1093/annonc/mdm496

[bib4] Di Fiore F, Blanchard F, Charbonnier F, Le Pessot F, Lamy A, Galais MP, Bastit L, Killian A, Sesboüé R, Tuech JJ, Queuniet AM, Paillot B, Sabourin JC, Michot F, Michel P, Frebourg T (2007) Clinical relevance of KRAS mutation detection in metastatic colorectal cancer treated by cetuximab plus chemotherapy. Br J Cancer 96: 1166–1169 17375050 10.1038/sj.bjc.6603685 PMC2360149

[bib5] Frattini M, Saletti P, Romagnani E, Martin V, Molinari F, Ghisletta M, Camponovo A, Etienne LL, Cavalli F, Mazzucchelli L (2007) PTEN loss of expression predicts cetuximab efficacy in metastatic colorectal cancer patients. Br J Cancer 97: 1139–1145 17940504 10.1038/sj.bjc.6604009 PMC2360431

[bib6] Khambata-Ford S, Garrett CR, Meropol NJ, Basik M, Harbison CT, Wu S, Wong TW, Huang X, Takimoto CH, Godwin AK, Tan BR, Krishnamurthi SS, Burris III HA, Poplin EA, Hidalgo M, Baselga J, Clark EA, Mauro DJ (2007) Expression of epiregulin and amphiregulin and K-ras mutation status predict disease control in metastatic colorectal cancer patients treated with cetuximab. J Clin Oncol 25: 3230–3237 17664471 10.1200/JCO.2006.10.5437

[bib7] Lièvre A, Bachet JB, Boige V, Cayre A, Le Corre D, Buc E, Ychou M, Bouché O, Landi B, Louvet C, André T, Bibeau F, Diebold MD, Rougier P, Ducreux M, Tomasic G, Penault-Llorca F, Laurent-Puig P (2008) KRAS mutations as an independent prognostic factor in patients with advanced colorectal cancer treated with cetuximab. J Clin Oncol 26: 374–379 18202412 10.1200/JCO.2007.12.5906

[bib8] Lièvre A, Bachet JB, Le Corre D, Boige V, Landi B, Emile JF, Côté JF, Tomasic G, Penna C, Ducreux M, Rougier P, Penault-Llorca F, Laurent-Puig P (2006) KRAS mutation status is predictive of response to cetuximab therapy in colorectal cancer. Cancer Res 66: 3992–3995 16618717 10.1158/0008-5472.CAN-06-0191

[bib9] Park HW, Kang HC, Kim IJ, Jang SG, Kim K, Yoon HJ, Jeong SY, Park JG (2007) Correlation between hypermethylation of the RASSF2A promoter and K-ras/BRAF mutations in microsatellite-stable colorectal cancers. Int J Cancer 120: 7–12 17013898 10.1002/ijc.22276

